# Adverse Events Reporting Quality of Randomized Controlled Trials of COVID-19 Vaccine Using the CONSORT Criteria for Reporting Harms: A Systematic Review

**DOI:** 10.3390/vaccines10020313

**Published:** 2022-02-17

**Authors:** Cindra Tri Yuniar, Bhekti Pratiwi, Ardika Fajrul Ihsan, Bambang Tri Laksono, Iffa Risfayanti, Annisa Fathadina, Yeonseon Jeong, Eunyoung Kim

**Affiliations:** 1 Department of Pharmacology and Clinical Pharmacy, School of Pharmacy, Institut Teknologi Bandung, Bandung 40132, Indonesia; cindra@fa.itb.ac.id (C.T.Y.); bhekti@fa.itb.ac.id (B.P.); fajrul@fa.itb.ac.id (A.F.I.); bambang.tl@fa.itb.ac.id (B.T.L.); iffarisfa@students.itb.ac.id (I.R.); annisafathadina@students.itb.ac.id (A.F.); 2 Clinical Data Analysis, Evidence-Based Clinical Research Laboratory, Department of Health Science & Clinical Pharmacy, College of Pharmacy, Chung-Ang University, Seoul 06974, Korea; yysuni72@cau.ac.kr

**Keywords:** adverse event, harm, COVID-19 vaccine, systematic review, randomized controlled trial

## Abstract

Background: Assessing the quality of evidence from vaccine clinical trials is essential to ensure the safety and efficacy of the vaccine and further enhance public acceptance. This study aims to summarize and critically evaluate the quality of harm reporting on randomized controlled trials for the COVID-19 vaccine and determine the factors associated with reporting quality. Methods: We systematically searched the literature using PRISMA guidelines for randomized controlled trials (RCT) on COVID-19 Vaccine until 30 December 2021. Published articles were searched from electronic databases such as PubMed, Science Direct, Google Scholar, and Bibliovid. Bias analysis was performed using RoB-2 tools. The quality of reporting was assessed by the Consolidated Standards of Reporting Trials (CONSORT) harm extension modified into 21 items. Results: A total of 61 RCT studies (402,014 patients) were analyzed. Over half the studies demonstrated adequate reporting (59.02%), and 21 studies (34.4%) reported a low risk of bias. All studies reported death and serious adverse events (AEs), but only six studies mentioned how to handle the recurrent AEs. Reporting of AEs in subgroup analysis was also poor (25%). Conclusion: The RCTs on the COVID-19 vaccine were less biased with good quality on reporting harm based on the modified CONSORT harm extension. However, study quality must be considered, especially for a balance of information between effectivity and safety.

## 1. Introduction

On 30 January 2020, the WHO declared the COVID-19 disease outbreak as a public health emergency of international concern [1]. The emerging pandemic triggered researchers to find and develop some strategies to tackle the outbreak. Based on clinical trials, various drugs have been reported to be an effective therapy for COVID-19 infections. On the other hand, rapid diagnosis and an effective COVID-19 vaccine will complement the therapy and control the epidemic aroused by emerging viruses [2]. As announced by the WHO, there are now 284 COVID-19 vaccines under development, of which 104 are in clinical trials [3].

The first COVID-19 vaccination took place on 14 December 2020, in the US [4]. To date, almost half of the world population (47.8%) has received at least one dose COVID-19 vaccine [5]. Vaccine safety and effectiveness remain a public concern throughout the world [6]. Vaccine acceptance in low- and middle-income countries (LMICs) are primarily explained by an interest in personal protection against COVID-19, while concerns about side effects are the most common reason for hesitancy [7].

The European Medical Agency (EMA) reviewed 18 cases of unique thromboembolic events in combination with thrombocytopenia, such as cerebral venous sinus thrombosis (CVST), on 19 April 2021 [8]. In early April, the EMA safety committee confirmed unusual blood clots as very rare side effects of the COVID-19 Vaccine by AstraZeneca [9]. Other cases were reported in the US regarding the CVST event following the Ad26.COV2.S COVID-19 vaccine (Johnson&Johnson, Amersfoort, The Netherlands). There were 12 cases of CVST with thrombocytopenia that represent serious events from 2 March to 21 April 2021 [10]. Other adverse effects after mRNA vaccination BNT162b2 (Pfizer-BioNTech, Mainz, Germany) or mRNA-1273 (Moderna, Cambridge, MA, USA) were evaluated in the United States and Israel [11,12]. These studies reported the events that occurred during 21 days post-vaccination. A study in the US reported the incidence of ischaemic stroke, appendicitis, and acute myocardial infarction [11]. On the other hand, data from Israel’s largest health care organization showed that the vaccination was most strongly associated with an elevated risk of myocarditis, lymphadenopathy, appendicitis, and herpes zoster infection [12]. These events can usually be seen during the clinical trial, but rare cases may occur after launch. These case reports give rise to a negative perception about the COVID-19 vaccine and discourage some countries from implementing the program.

The best source of information about the effects of medical interventions, including vaccines, can be obtained through randomized controlled trials (RCTs). The benefit is usually measured in RCTs, with harms usually included as secondary outcomes. The important role of RCTs in a medical intervention encourage authors to present sufficient and appropriate details regarding both benefit and harms in the report [13].

Transparent and accurate reporting of trial data is equally important in addition to robust trial design, especially reporting of harm data related to interventions 3. In 2004, the Consolidated Standards of Reporting Trials (CONSORT) Group produced an extension to its guidelines for reporting trial results to include the reporting of harms, but these guidelines are poorly implemented in practice [14]. The adverse events (AE) collection and presentation method highlighted inadequacies and inconsistencies in AE reporting described in various published clinical trials [3,14].

Assessing the quality of evidence from vaccine clinical trials is also essential to ensure the safety and efficacy of the vaccine and further enhance public acceptance. Despite the increasing number of clinical trials assessing vaccine strategies for COVID-19, these trials’ harm reporting quality remains questionable. This study aims to summarize and critically evaluate the quality of harm reporting on a randomized controlled trial in the COVID-19 vaccine and define the determinant factors associated with reporting quality.

## 2. Materials and Methods

### 2.1. Search Strategy and Selection Criteria

This study used The Preferred Reporting Items for Systematic Reviews and Meta-Analyses (PRISMA) guideline for the study design, search protocol, screening, and reporting. Published articles were searched from electronic databases: PubMed, Science Direct, Google Scholar, and Bibliovid. The search strategy included all terms related to COVID-19, SARS-CoV-2, and COVID-19 Vaccine Trial. Electronic databases were originally searched from inception until 30 December 2021.

Two independent reviewers (CTY, BP) conducted a review of titles, abstracts, and full papers. Ten of the included RCTs were tested for a pilot analysis wherein two authors (CTY and BP) independently analyzed the trials using the checklist of CONSORT harm. If disagreements among the test RCTs existed, two reviewers discussed to obtain consensus and, if needed, a third reviewer (EYK) acted as the arbitrator [13,14]. This pilot test was repeated twice to ensure inter-rater agreement. The Cohen’s kappa metric was used for measuring agreement between the two authors using a previously described methodology [14,15]. The inclusion criteria were randomized controlled trials published articles evaluating the COVID-19 vaccine in English. Studies were excluded if they were (1) non-randomized, observational studies, animal studies, reviews, case reports, or letter, and (2) duplicate publications.

### 2.2. Risk of Bias Assessment

Two authors independently assessed the risk of bias using the Cochrane risk-of-bias tool for randomized trials second version (RoB-2) for included studies. This tool is a standardized method for assessing potential bias in reports of randomized interventions, consisting of a fixed set domain of bias due to randomization process, deviations from intended interventions, missing outcome data, measurement of the outcomes, and selection of reported results. A proposed judgment about the risk of bias arising from each domain is generated by an algorithm, where judgment can be “low” or “high” risk of bias or can express “some concerns” [3,16].

### 2.3. Quality of Reporting of Harm Data

Data on harm reporting from all included trials were assessed by two authors using CONSORT Extension of Harms 2004, a 10-item checklist [14]. A single parameter may be misleading and be challenging to define; therefore, we adapted the methodology used by previous studies and modified it into a 21-item checklist [3,13,14,15]. Each item of the 21-item checklist was scored individually and weighted with equal importance in line with CONSORT harm recommendations. If it were adequately reported on the paper, we would put a score of 1. On the contrary, the 0 score carries if it was inadequately reported or not reported at all. Then, the total score was calculated by summation of all individual scores, called Total Harm Reporting Score (THRS). THRS% was then calculated by dividing the number of items met by the number of total items to generate a percentage score. The adherence to CONSORT harm recommendation is concluded from a percentage more than or equal to 70%. Adherence 70% and above was defined as adequate and below 70% as inadequate [17].

### 2.4. Data Extraction and Analysis

All included studies were extracted onto a summary table with the following information: journal impact factor, number of authors, study design, number of trial subjects, type of intervention (vaccine and control), dosage of vaccine, age, gender, baseline severity, effectiveness indicator, serious and total adverse events. We used descriptive statistics on the characteristics of RCT studies. The overall number and proportion (%) of RCT that met each of the CONSORT for harm checklist items were determined.

### 2.5. Statistical Analysis

Cohen’s kappa (point estimate) was determined to appraise inter-rater agreement per CONSORT harm item between two-authors analysis. A kappa point estimate between 0.60 and 0.80 was considered indicative of substantial agreement, while a figure above 0.80 was appraised as an almost perfect agreement [15].

A univariate and multivariate linear regression analysis was planned to determine the possible determinants for high THRS. The dependent variable was THRS of CONSORT harm item checklist. The independent variables were journal impact factors (≥50, <50), number of authors (<20, 20–50, >50), number of subjects (sample size), phase of clinical trials (phase 1, 2, 3, 4), type of study (single-center, multi-center), funding status (non-industry, industry), and participant flowcharts (yes, no). Odds ratios (ORs) and confidence intervals (95% CIs) obtained from the analysis are also presented. All tests for statistical significance were two-tailed, with the threshold set at 0.05. All analyses were performed using SPSS software (version 26.0; IBM Corporation, Armonk, NY, USA).

## 3. Results

### 3.1. Result of the Search

**Figure 1:**
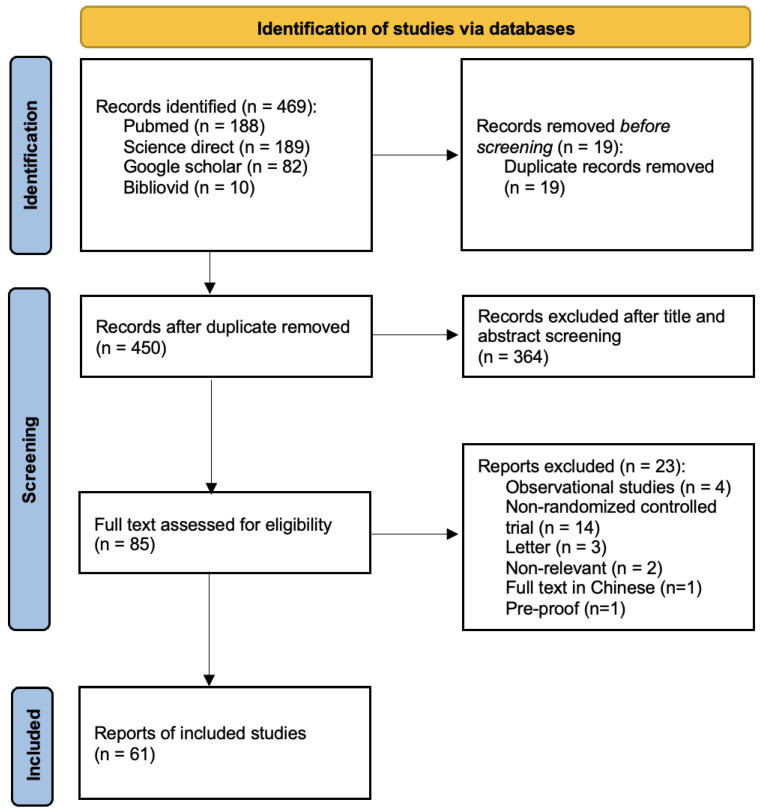
Preferred Reporting Items for Systematic Reviews and Meta-Analyses (PRISMA) flow diagram of the study selection process.

From the original search strategy and selection process, 469 studies were identified. After screening and implementation of inclusion criteria, 61 randomized controlled trials (RCTs) were eligible and included in this review, with the total number of subjects about 402,014 [18,19,20,21,22,23,24,25,26,27,28,29,30,31,32,33,34,35,36,37,38,39,40,41,42,43,44,45,46,47,48,49,50,51,52,53,54,55,56,57,58,59,60,61,62,63,64,65,66,67,68,69,70,71,72,73,74,75,76,77,78]. Data were extracted from these 61 clinical trials (Figure 1).

### 3.2. Study Characteristics

Forty studies met the criteria for inclusion in this review. A summary of the characteristics of the included studies is provided in Table 1. The most common type of vaccine in these studies was viral vector, with the viral vectors used were adenovirus in 11 studies [17,22,23,26,29,33,34,36,39,40,54] and baculovirus in 2 studies [35,48]. Eighteen studies using vaccines with adjuvants: AlOH3/Algel-IMDG in 21 studies [18,19,20,27,28,29,30,31,35,45,48,51,52,55,56,57,59,64,66,68,70], Matrix-M1 in three studies [21,63,74], AS03/CpG + Alum in one study [25], Af03/AS03 in one study [36], and MF59 in one study [42].

The included studies were conducted in several countries, mostly in China (*n* = 19), the United States (*n* = 12), and the United Kingdom (*n* = 6). Most studies (62.5%) were multicenter studies. One study was conducted in Argentina, Brazil, Chile, Columbia, Mexico, Peru, South Africa, and United States [17], two studies were conducted in the United States, Argentina, Brazil, South Africa, Germany, and Turkey [24], one study was conducted in the United Kingdom, Brazil, and South Africa [34], one study was conducted in Belgium and United States [57], and one study was conducted in Germany and Belgium [47]. Most studies (*n* = 24) were double-blind studies. Only six studies were triple-blind studies [24,32,38,41,43,46].

Most studies (68.8%) were conducted in phase I, II, or I/II clinical trials. Only eight studies were conducted in phase III clinical trials [17,22,24,33,34,38,46,51]. Two studies were conducted in phase IV clinical trials [75,77]. The number of participants in these studies ranged from 44–44.325 participants. Twenty-three studies had more than 1000 participants. There are six studies with more than 20,000 participants [17,22,24,33,34,38]. The age of participants in the studies was in the range of 3–100 years old. Most studies (*n* = 16) had participants in the age range 18–60 years old (adults). Three studies had participants only in children and adolescents age [20,30,46] and one study in older age (more than 60 years old) [31].

### 3.3. Risk of Bias

**Figure 2:**
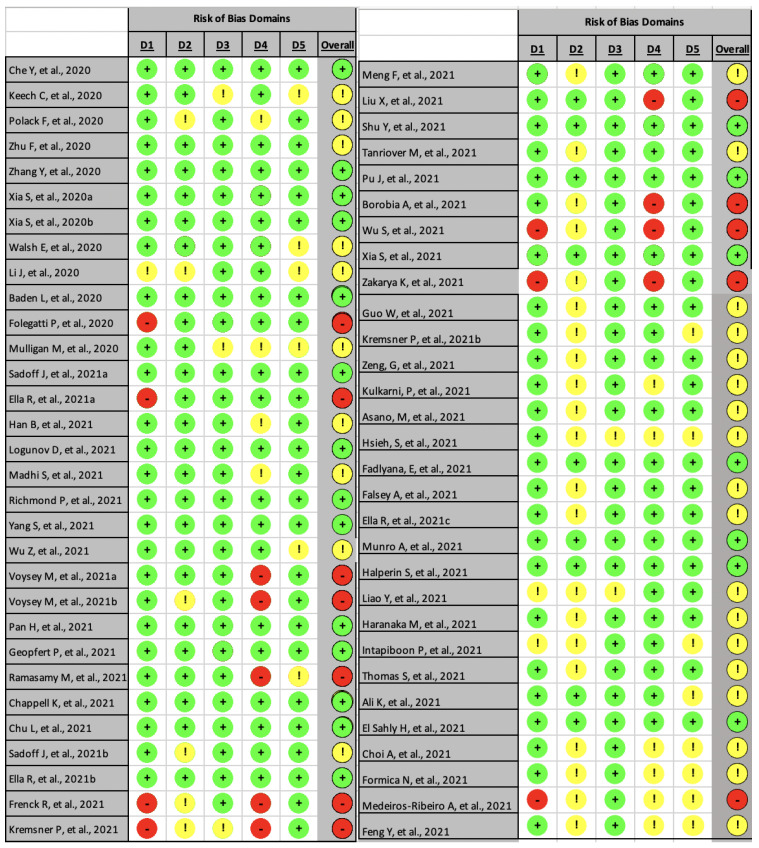
Summary of risk of bias analysis using ROB2 tools.

The risk of bias was analyzed using the RoB 2 tools for 61 trials included in this review. Among them, 12 clinical trials scored as “high-risk” [19,33,34,39,40,46,47,49,53,54,55,77]; 28 trials categorized as trials with ‘some concerns’; while the remaining trials (21 studies) were classified as “low-risk” (Figure 2). Four studies were identified with the most “high-risk” due to unconcealed intervention to the participants and outcome measure affected by the knowledge of investigators [46,47,54,55].

The most significant domain that induced the high-risk judgment was biased in measurement of the outcome (domain 4) [33,34,40,46,47,49,53,54,55]. There were concerns that the clinical trial outcome would be affected by prior knowledge of the outcome assessor. Another conclusion related to the high-risk judgment was the randomization process—as the prime-booster process in vaccination obligated participants to continue receiving the same vaccine type [34,53].

The second most significant domain that induced the ‘some concern’ was bias caused by deviations from the intended intervention (domain 2), in which 27 trials also appeared to have a probability of risk [24,33,37,44,46,47,48,51,53,54,55,58,59,60,62,64,66,67,68,69,70,73,74,75,76,77]. Almost all of the bias on domain 2 is because of disclosure of the intervention, whether to patients and or the carer of the patients. The risk could only cause “some concerns” if there was no information on whether the deviation was caused by the trial context [24,44,46,47,48,51,53,54,55]; or if the deviations were caused by trial context and possibly intervene the outcome but be balanced in each group [46].

Only five trials had “some concern” of missing outcome bias [22,42,48,59,64]. Most of the bias on domain 3 was caused by missing patient data, from patients withdrawing or losing contact and preventing a follow-up.

### 3.4. Adherence to CONSORT Recommendations

The total harm reporting score (THRS) and percentage of randomized controlled trials fulfilling the CONSORT Extension of Harms are items and inter-agreement (assessed by Cohen’s kappa) was 0.785. The median score for THRS was 15, with a minimum of 7 and a maximum of 20, of a possible total of 21. A total of 36 studies (59.02%) adhered with the CONSORT recommendation for reporting harms, with the two highest studies reporting 20 score of 21 THRS, as shown in Table 2.

The highest percentage is reported of deaths and serious adverse events (AEs). All of the studies reported serious adverse events (100%). The majority of the studies mentioned adverse events in the title or abstract (93%). Almost all studies used the safety term to define the adverse event in the title or abstract. Nearly all studies presented the timing of data collection of AE data (97%) and mentioned adverse events in the introduction part (92%). Most studies explained the method of presenting and/or analyzing AEs, severity, and grading of AEs, provided both numbers of adverse events and patients with adverse events, have a balanced discussion of benefit-harm and study limitation.

Only six studies (10%) provided an approach for the handling of recurrent AEs [29,47,55,58,59,74]. The authors rarely reported subgroup analysis and exploratory analysis for harms (25%), only 15 studies reported the subgroup analysis [17,22,24,32,36,37,40,50,58,60,68,70,71,73,74]. Less than half of the studies reported the plan for monitoring and rules for stopping the trial because of harms (31%), the withdrawal person on each harm (49%), and definition use for AEs analysis set (41%). A total of 85% reported using an active surveillance approach to collect AEs during the study, but only 64% of studies mentioned the person who was attributed to the trial drug. Most of the trials presented AEs separately for each arm (90%).

### 3.5. Determinant Factors of Reporting Quality

Potential factors attributing to the overall reporting quality were determined using linear regression analysis. In the univariate analysis, the number of authors (≥50) and type of study (multi-center) were associated significantly with the low THRS, with the score OR = −4.237, 95% CI −6.804 to -1.617 (*p* = 0.02) and OR = −1.462, 95% CI −2.281 to −0.102 (*p* = 0.036), respectively. In multivariate analysis, the number of authors (≥50) was also significantly associated in determining the low THRS (OR = −4.439, 95% CI −7.40 to −1.47). On the other hand, journal impact factor and funding status had a positive association with THRS, but was not statistically significant. The graph illustrating the exact relation between the number of authors, or funding status, or impact factors, and the CONSORT score shown in Supplementary Figure S2.

## 4. Discussion

This systematic review evaluated the randomized clinical trial for the COVID-19 Vaccine from inception to December 2021 and aimed to summarize and critically evaluate the quality of harm reporting and define the determinant factors associated with reporting quality. The rapid development of the COVID-19 vaccine triggered the industry and government (or research institute) to conduct a randomized clinical trial, starting from an inactivated vaccine. More than 90% of RCTs involved adult subjects (≥18 years old) due to having a prioritized area for COVID-19 transmission. As there was a high incidence of death in the elderly with COVID-19, the trials prioritized elderly subjects over adults. In the last term of data collection, several studies also started to conduct trials on child participants and vaccine boosters.

A total of 61 studies were included in this study, and more than half of the studies (59.02%) complied with CONSORT extension of harms. During the COVID-19 pandemic, the publisher gave free access to full-text articles to enhance the COVID-19 vaccine development, helping the authors in collecting the best literature available. Identifying studies as a randomized trial in the search database was also helped by titles that explicitly mentioned the content. Regarding the vaccine development in clinical trial phase 1 and/or 2, the published journal usually mentioned the safety assessment on the title or abstract so that the reported safety item is high. This result is in line with a previous review that concluded that a randomized controlled trial prospectively defined the intervention’s adverse effects and specified the detail of monitoring [79].

Deaths, serious AEs, and events leading to discontinuation of intervention should always be reported for the safety assessment in the trial [80]. In line with that, the highest percentage of CONSORT harm items was reported deaths and serious AEs (item 6b). All the studies reported death and serious adverse events; despite having no events (0 cases) in the study, the trials mentioned no serious events or death related to vaccination in the text. The adverse events reported from randomized clinical trials were limited to the number of participants and study setting. Interindividual variability might affect the events on a specific vaccine. Moreover, specific adverse events are not entirely captured despite occurring in trials; thus, adverse event reporting transparency is essential. We summarized the adverse events reported in included studies in Supplementary Material Table S3.

Interindividual variation may be observed in subgroup analysis. Subgroup analysis was defined as reporting any subgroup effect. Among 61 studies, only 15 studies (25%) reported subgroup analysis. This result aligns with a previous study that showed suboptimal reporting of subgroup analysis in high-impact journals. Trials funded by the industry may more commonly report this aspect, but the authors analyzed using inappropriate methods [81]. The lack of subgroup reporting will affect the physician-decision making and also the public acceptance of vaccination program.

The item related to the recurrent events reporting was poor when analyzing all parameters. Events reported in trials are usually defined as cases, and the number of events is counted by how many cases occur on participants. Only six studies clearly mentioned how they measured and counted cases as events and how they handled them—crude incidence rates or exposure-adjusted incidence rates, which were often analyzed on randomized clinical trials. For individual patient’s profiles, these rates do not adequately account the events because the opportunity of multiple events occurs during the trial. Some AEs were also possible to correlate with other AEs or might lead to another AEs in the future [82].

Although the journal impact factor is not associated significantly with compliance, the high THRS of CONSORT harm reporting recommendation was dominantly reported on high impact journals (≥50). Compared with *NEJM* and *JAMA*, *The Lancet* are the only journals that asked authors to provide and follow CONSORT harm extension in the guidelines [79]. Adherence to CONSORT for abstract checklist is also high in *The Lancet*, compared with *NEJM*, *Annals IM*, *The BMJ*, and *JAMA* [83]. Indeed, the requirement from journals will enhance the reporting quality of harm on RCTs.

Among all independent parameters, the number of authors was statistically significant with the THRS based on univariate and multivariate analysis. The other parameters (sample size, clinical phase study, participant flowchart, type of study) had a negative association with THRS, except for journal impact factor and funding status. Journal impact factors was not significantly associated with THRS due to many publications for COVID-19 vaccine trials usually published in high impact factors journal (73.8%), despite high-impact journals commonly limiting the number of authors.

Univariate analysis showed that more authors involved in the manuscript would lead to low adherence on RCTs harm reporting. At the same time, studies with many authors (>50) are usually due to it being a multi-center study. Funding from the industry also was positively associated with the high THRS. This result might correlate with the industry standard in COVID-19 vaccine development and ensure the safety aspects in RCTs fulfill the authorization criteria.

To the best of our knowledge, the present study is the first to evaluate the harm reposting quality using CONSORT harm extension on the COVID-19 vaccine trial. We also include an evaluation from various databases and use PRISMA methods. Furthermore, we also determine the factors associated with adequate reporting quality. This review suggests the authors/company who are reporting the RCT should comply with the standardized reporting of trials and harm-related vaccines. Since the adverse event is usually pointed out by the public, good quality of reporting of harm will give clear and complete information about adverse events for specific Vaccine products. We hope our result will enhance public trust in COVID-19 Vaccine RCTs. The RCT studies evaluating the safety and efficacy of COVID-19 Vaccine to special populations such as those with autoimmune disease are rarely included in this study due to a discrepancy in the inclusion and exclusion criteria. In future, more studies including special groups are expected. This study has assessed the quality of harm reporting on COVID-19 vaccines RCTs. In the future, studies on overall reporting quality with efficacy and outcome measures are required to balance information between efficacy and safety.

## 5. Conclusions

It is concluded that the RCTs on COVID-19 vaccine were less biased in RoB analysis, and more than half of the studies fulfill the CONSORT harm extension. However, the quality of the study needs to be considered, especially the balance of information between effectivity and safety reasons; this will affect the public acceptance and policy on COVID-19 vaccinations.

## Figures and Tables

**Table 1:** Characteristics of Eligible Studies.

Study Characteristic	No. (%)
Total, No.	61
Type of vaccine	
Inactivated vaccine	18 (29.5)
mRNA	18 (29.5)
Protein subunit	7 (11.5)
Viral vector	18 (29.5)
No. of subjects	
1–500	22 (36.1)
500–1000	16 (26.2)
>1000	23 (37.3)
Age of participants	
<18	4 (6.6)
≥12	1 (1.7)
≥18	56 (96.7)
Type of study	
Multi-center	35 (57.4)
Single-center	26 (42.6)
Study design	
Open-label	6 (9.8)
Single-blind	15 (24.6)
Double-blind	33 (54.1)
Triple blind	6 (9.8)
Not stated	1 (1.7)
Clinical trial phase	
Phase I	11 (18)
Phase I/II	19 (31.1)
Phase II	12 (19.7)
Phase II/III	4 (6.6)
Phase III	9 (14.7)
Phase I–III	4 (6.6)
Phase IV	2 (3.3)
Impact Factor	
<50	45 (73.8)
≥50	16 (26.2)
Number of authors	
<20	16 (26.2)
20–50	40 (65.6)
>50	5 (8.2)
Funding	
Industry	31 (50.8)
Non-industry	30 (49.2)
Participants flowchart	
Yes	54 (88.5)
No	7 (11.5)

**Table 2:** Items fulfilled against the quality of reporting criteria (Consolidated Standards of Reporting Trials (CONSORT) Extension for Harm) (*n* = 61).

Section of Paper	CONSORT Harm Recommendation	Detailed Items	Compliance of Trials, *n* (%)
Title and abstract	1. If the study collected data on harms and benefits, the title or abstract should state so	1. Adverse events mentioned in title or abstract	57 (93)
Introduction	2. If the trial addresses both harms and benefits, the introduction should state so	2. Information on adverse events mentioned in the introduction	56 (92)
Methods	3. Include a list of AEs with definitions for each (with attention, when relevant, to grading, expected vs. unexpected events, references to standardized and validated definitions, and description of new definitions	3a. Definitions of AEs mentioned	50 (82)
		3b. If article mentioned all or selected sample of AE	45 (74)
		3c. If article mentioned the use of a validated instrument to report adverse event severity	25 (41)
	4. Clarify how harms-related information was collected (mode of data collection, timing, attribution methods, intensity of ascertainment, and harms-related monitoring and stopping rules, if pertinent)	4a. Describe the mode of data collection (e.g., diaries, phone interviews, face-to-face interviews)	52 (85)
		4b. Stated the timing of collection of AE data	59 (97)
		4c. Description of how AE were attributed to trial drugs	39 (64)
		4d. Described the plan for monitoring for harms and rules for stopping the trial because of harms	19 (31)
	5. Describe plans for presenting and analyzing information on harms (including coding, handling of recurrent events, specification of timing issues, handling of continuous measures, and any statistical analyses)	5a. Described the methods for presenting and/or analyzing adverse events	53 (87)
		5b. Description of approach for the handling of recurrent AEs	6 (10)
Result	6. Describe for each arm the participant withdrawals that are due to harm and the experience with the allocated treatment	6a. Reported withdrawals because of AE in each arm	30 (49)
		6b. Reported deaths and serious AEs	61 (100)
	7. Provide denominators for describing harms	7a. Provided denominators for adverse events	44 (72)
		7b. Provided definitions used for analysis set (intention to treat, per protocol, safety data available, unclear)	25 (41)
	8. Present the absolute risk of each adverse event (specifying type, grade, and seriousness per arm), and present appropriate metrics for recurrent events, continuous variables, and scale variables, whenever pertinent	8a. Reported results separately for each treatment arm	55 (90)
		8b. Severity and grading of AEs	56 (92)
		8c. Provided both number of adverse events and number of patients with adverse events	57 (93)
	9. Describe any subgroup analysis and exploratory analysis for harms	9. Described subgroup analysis and exploratory analysis for harms	15 (25)
Discussion	10. Provide a balanced discussion of benefits and harms with emphasis on study limitations, generalisability and other sources of information on harms	10a. Provided a balanced view that puts benefits and harms into perspective	55 (90)
		10b. Included limitations of study with respect to harms (e.g., lack of power, short duration of exposure, inconclusive findings, post hoc analysis, generalisability of AE info as dependent on clinical setting)	57 (93)

## Data Availability

The data presented in this study are available on this paper. The data were collected from published data.

## Supplementary Materials

The following are available online at https://www.mdpi.com/article/10.3390/vaccines10020313/s1, Table S1: PRISMA 2020 checklist for systematic review, Table S2: Search Terms Used, Table S3. Characteristics of COVID-19 Randomized control trials included in this study, Figure S1: Chart of Trial for Item Fulfilled, Figure S2: Graph for the relationship of RCTs with the number of authors, funding status, and impact factor.
