# Supplementary material for: Adverse Events Reporting Quality of Randomized Controlled Trials of COVID-19 Vaccine Using the CONSORT Criteria for Reporting Harms: A Systematic Review
Source: Vaccines (Basel). 2022 Feb 17;10(2):313. doi: 10.3390/vaccines10020313 (PMC8875800; doi:10.3390/vaccines10020313)
Supplement: Supplementary file 1 [file vaccines-10-00313-s001.zip › Figure S2.pdf]

Supplementary Figure S2

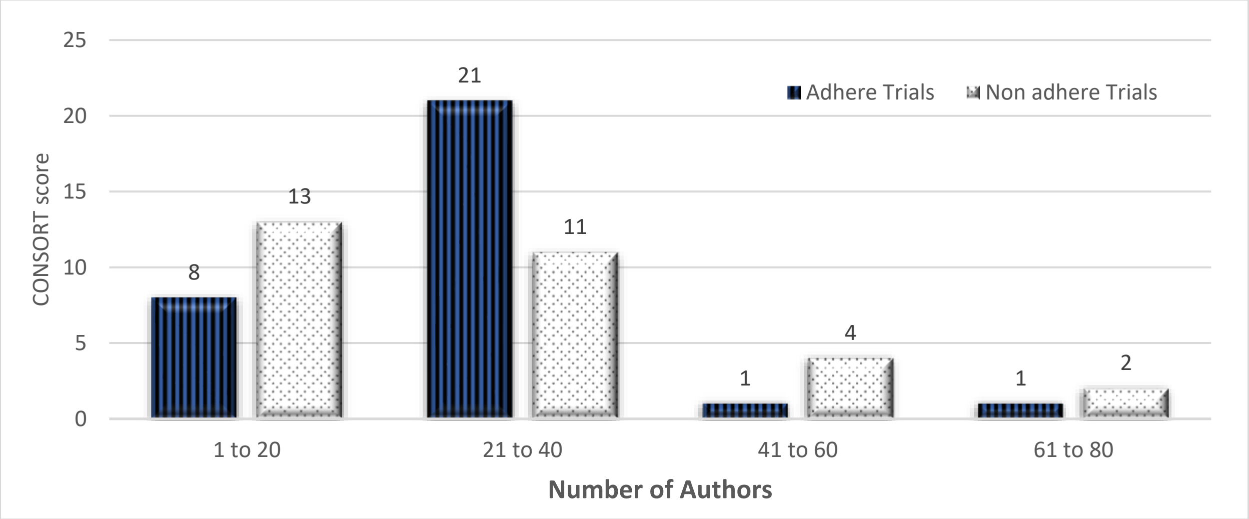

(a). Number of authors and Adherence to CONSORT harm score recommendation

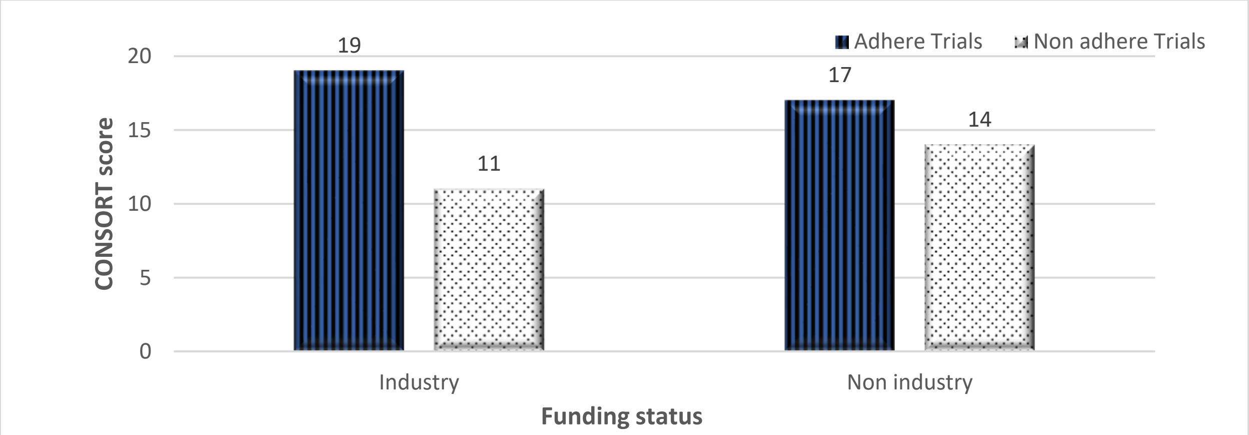

(b). Funding status and Adherence to CONSORT harm recommendation

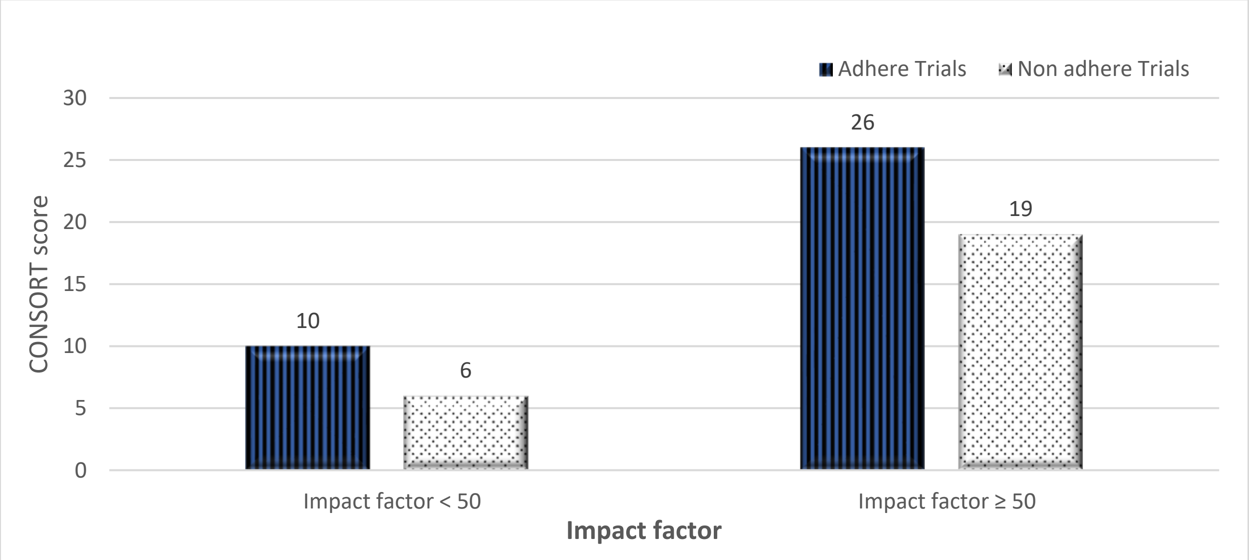

(c). Journal impact factor and Adherence to CONSORT harm recommendation
