# Supplementary material for: Adverse Events Reporting Quality of Randomized Controlled Trials of COVID-19 Vaccine Using the CONSORT Criteria for Reporting Harms: A Systematic Review
Source: Vaccines (Basel). 2022 Feb 17;10(2):313. doi: 10.3390/vaccines10020313 (PMC8875800; doi:10.3390/vaccines10020313)
Supplement: Supplementary file 1 [file vaccines-10-00313-s001.zip › Table S2.pdf]

SEARCH TERMS USED FOR GATHERING DATA

No	Database	Keywords	Note
1	Pubmed	covid-19 OR SARS-Cov-2, AND vaccine, AND trial	
2	Science direct	covid 19 vaccine trial	advanced search on title keyword
3	Google scholar	covid 19 vaccine trial OR SARS-Cov-2 vaccine trial	
4	Bibliovid	covid 19 vaccine trial	
