# Supplementary figures and images for: Adverse Events Reporting Quality of Randomized Controlled Trials of COVID-19 Vaccine Using the CONSORT Criteria for Reporting Harms: A Systematic Review
Source: Vaccines (Basel). 2022 Feb 17;10(2):313. doi: 10.3390/vaccines10020313 (PMC8875800; doi:10.3390/vaccines10020313)

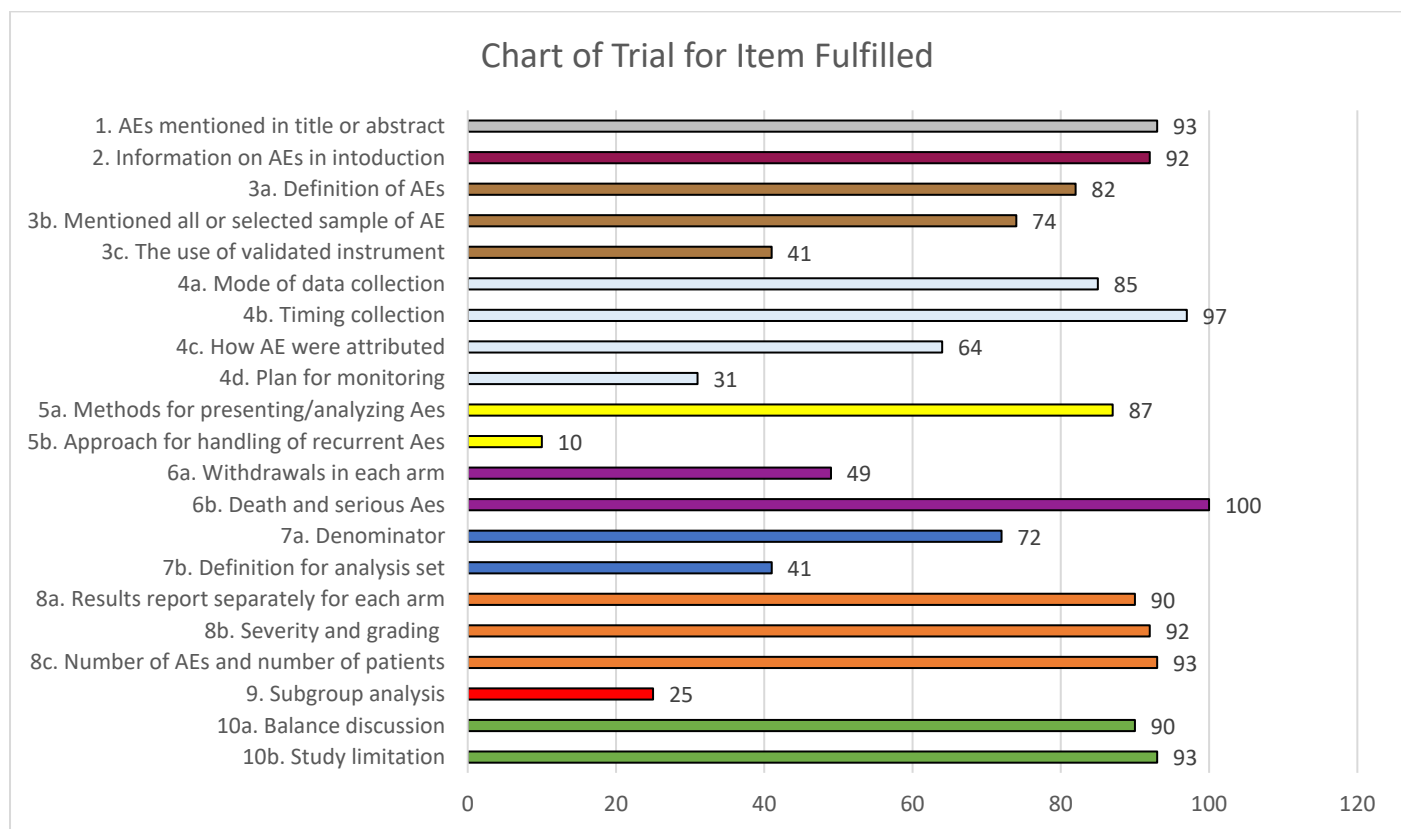

**Supplementary Figure S1. Chart of Trial for Item Fulfilled (N=61)**

Supplement: Supplementary file 1 [file vaccines-10-00313-s001.zip › Figure S1.pdf]
